# A Novel NMDA Receptor Antagonist Protects against Cognitive Decline Presented by Senescent Mice

**DOI:** 10.3390/pharmaceutics12030284

**Published:** 2020-03-22

**Authors:** Júlia Companys-Alemany, Andreea L. Turcu, Aina Bellver-Sanchis, Maria I Loza, José M. Brea, Anna M Canudas, Rosana Leiva, Santiago Vázquez, Mercè Pallàs, Christian Griñán-Ferré

**Affiliations:** 1 Pharmacology Section, Department of Pharmacology, Toxicology and Therapeutic Chemistry, Faculty of Pharmacy and Food Sciences, Institute of Neuroscience, University of Barcelona (NeuroUB), Av. Joan XXIII 27-31, 08028 Barcelona, Spain; juliacompanysalemany@gmail.com (J.C.-A.); abellver@gmail.com (A.B.-S.); canudas@ub.edu (A.M.C.); christian.grinan@ub.edu (C.G.-F.); 2 Laboratori de Química Farmacèutica (Unitat Associada al CSIC), Department de Farmacologia, Toxicologia i Química Terapèutica, Facultat de Farmàcia i Ciències de l’Alimentació, and Institute of Biomedicine (IBUB), Universitat de Barcelona, Av. Joan XXIII, 27-31, 08028 Barcelona, Spain; aturcu@ub.edu (A.L.T.); rosana.leiva58@gmail.com (R.L.); svazquez@ub.edu (S.V.); 3 Innopharma Screening Platform, Biofarma Research Group, Centro de Investigación en Medicina Molecular y Enfermedades Crónicas (CIMUS), Universidad de Santiago de Compostela, 15701 Santiago de Compostela, Spain; Mabel.loza@usc.es (M.I.L.); pepo.brea@usc.es (J.M.B.)

**Keywords:** NMDAR antagonist, cognitive decline, neurodegeneration, aging, Alzheimer’s disease, oxidative stress, BDNF, apoptosis

## Abstract

Alzheimer’s disease (AD) is the leading cause of dementia. Non-competitive N-Methyl-D-aspartate (NMDA) receptor antagonist memantine improved cognition and molecular alterations after preclinical treatment. Nevertheless, clinical results are discouraging. In vivo efficacy of the RL-208, a new NMDA receptor blocker described recently, with favourable pharmacokinetic properties was evaluated in Senescence accelerated mice prone 8 (SAMP8), a mice model of late-onset AD (LOAD). Oral administration of RL-208 improved cognitive performance assessed by using the three chamber test (TCT), novel object recognition test (NORT), and object location test (OLT). Consistent with behavioural results, RL-208 treated-mice groups significantly changed NMDAR2B phosphorylation state levels but not NMDAR2A. Calpain-1 and Caspase-3 activity was reduced, whereas B-cell lymphoma-2 (BCL-2) levels increased, indicating reduced apoptosis in RL-208 treated SAMP8. Superoxide Dismutase 1 (SOD1) and Glutathione Peroxidase 1 (GPX1), as well as a reduction of hydrogen peroxide (H_2_O_2_), was also determined in RL-208 mice. RL-208 treatment induced an increase in mature brain-derived neurotrophic factor (mBDNF), prevented Tropomyosin-related kinase B full-length (TrkB-FL) cleavage, increased protein levels of Synaptophysin (SYN) and Postsynaptic density protein 95 (PSD95). In whole, these results point out to an improvement in synaptic plasticity. Remarkably, RL-208 also decreased the protein levels of Cyclin-Dependent Kinase 5 (CDK5), as well as p25/p35 ratio, indicating a reduction in kinase activity of CDK5/p25 complex. Consequently, lower levels of hyperphosphorylated Tau (p-Tau) were found. In sum, these results demonstrate the neuroprotectant role of RL-208 through NMDAR blockade.

## 1. Introduction

The prevalence of behavioural abnormalities associated with age-related cognitive decline is growing in older people [1]. Age is the most important risk factor for cognitive impairment and dementia because of natural changes like neuronal death or functional impairments [2]. Alzheimer’s disease (AD) is the most common type of dementia, affecting around 50 million people worldwide in 2018 [3]. A three-fold increase is estimated for the number of cases of AD to 131.5 million by 2050 [4,5]. The disease involves the degeneration of some areas of the brain, mainly the hippocampus, which results in behavioural changes, memory loss, and a decline in cognition functions [6]. Nevertheless, the causes of AD remain unknown, and no preventive or curative treatments are available.

The neuropathological hallmarks comprise the accumulation and deposition of β-amyloid (Aβ) in the senile plaques (SNPs). Likewise, the hyperphosphorylation of Tau protein is implicated in neurofibrillary tangles increase (NFTs) [7]. There are other important pathological processes under AD development. On one hand, oxidative stress (OS) [8], neuroinflammation [9], apoptosis [10], and synaptic abnormalities [11] play an essential role in the etiology of AD. On the other hand, there is accumulating evidence that several neurotransmitter pathways, such as acetylcholine, dopamine, glutamate, and serotonin [12] are involved in the pathological alterations of AD [13,14]. Indeed, glutamate-mediated toxicity is also one of the main processes responsible for memory impairment in AD. 

Glutamate is the primary excitatory neurotransmitter in the central nervous system (CNS), and it plays a critical role in cognitive functions [15]. N-Methyl-D-aspartate receptor (NMDAR) is an important subtype of ionotropic glutamate receptors, essential for the normal function of the CNS [16]. Under normal conditions, extracellular Mg2+ that allows Ca^2+^ to move into the cell for the following physiological functions gates NMDAR. Hence, glutamatergic neurotransmission through NMDAR is critical for neuroplasticity [14], neuronal survival [17], and learning and memory formation [18]. However, excessive levels of glutamate lead to the overactivation of NMDAR and allow a higher amount of Ca^2+^ influx into the nerve cell, NMDAR overactivation being a feature present in several brain disorders [19]. This excessive activity causes excitotoxicity and promotes neuronal loss, OS production [20], neuroinflammation and increases p-Tau [21]. Indeed, several studies have demonstrated the association between changes in the NMDAR levels in the cerebral cortex and hippocampus, and cognitive deficits, including anxiety and fear behaviour [22]. In sum, a potential implication for NMDAR in different aspects of the cognitive decline occurred in AD [21].

Because of the importance of NMDAR, several uncompetitive antagonists have been tested both in animal studies and in clinical trials [23]. However, most of the compounds tested have failed due to reduced tolerance and efficacy [24,25]. One possibility for this high rate of attrition was because these compounds blocked the physiological activity of glutamate-mediated by NMDAR activation [26], producing unacceptable side effects, such as psychosis and nausea, among others [27]. 

Memantine is an uncompetitive and well-tolerated NMDAR antagonist, which has been used to treat moderate to severe AD [28]. However, memantine possesses limited clinical efficacy [29]. Considering this, new moderate-affinity NMDAR antagonists with similar but distinct pharmacological properties are of interest. Recently, we have developed a novel polycyclic amine, RL-208, a voltage-dependent, moderate-affinity, uncompetitive NMDAR blocker characterized pharmacologically and electrophysiologically through in vitro approaches [30]. 

In the current study, we aimed to have the in vivo proof of concept determining the beneficial effect of RL-208 treatment in behavioural abnormalities and cognitive decline in a mouse model of aging and AD, the senescence-accelerated mouse prone 8 (SAMP8). Several molecular pathways related to NMDAR activation, apoptosis, OS neurotrophic support, and tau pathology characteristic for SAMP8 were also studied.

## 2. Materials and Methods

### 2.1. Reagents

RL-208, (3,4,8,9-tetramethyltetracyclo [4.4.0.0^3,9^.0^4,8^]dec-1-yl)methylamine hydrochloride was synthesized as previously described [30].

### 2.2. Pharmacological Characterization of RL-208

#### 2.2.1. Microsomal Stability in Human, Rat and Mice Microsomes

The human, rat, and mice microsomes employed were purchased from Tebu-Xenotech. The compound was incubated at 37 °C with the microsomes in a 50 mM phosphate buffer (pH = 7.4) containing 30 mM MgCl_2_, 10 mM NADP, 100 mM glucose-6-phosphate and 20 U/mL glucose-6-phosphate-dehydrogenase. Samples (75 µL) were taken from each well at 0, 10, 20, 40, and 60 min and transferred to a plate containing 4 °C 75 µL acetonitrile. Then, 30 µL of 0.5% formic acid in water was added to improve the chromatographic conditions. The plate was centrifuged (46,000× g, 30 min) and supernatants were taken and analyzed in a UPLC-MS/MS (Xevo-TQD, Waters) by employing a BEH C18 column and an isocratic gradient of 0.1% formic acid in water: 0.1% formic acid acetonitrile (60:40) for 5 minutes and a flow of 0.25 mL/min. The metabolic stability of the compounds was calculated from the logarithm of remaining compounds at each time point studied.

#### 2.2.2. Cytochrome Inhibition

To screen the inhibition potential of the compounds, recombinant human cytochrome P450 enzymes (CYP1A2, CYP2C9, CYP2C19, CYP2D6, and CYP3A4) and probe substrates were used with the fluorescent detection method. 

Incubations were conducted in 200-µL volume 96 well microtiter plates (COSTAR 3915). Addition of cofactor-buffer mixture (KH_2_PO_4_ buffer, 1.3 mM NADP+, 3.3 mM MgCl_2_, 3.3 mM Glucose-6-phosphate and 0.4 U/mL Glucose-6-phosphate Dehydrogenase), supersomes control, standard inhibitors (Furafyline, Tranylzypromine, Ketoconazole, Sulfaphenazole, and Quinidine; from Sigma Aldrich), and test compounds to plates were carried out using a a liquid handling station (Zephyr Caliper). The plate was then pre-incubated at 37 °C for 5 min, and the reaction initiated by the addition of pre-warmed enzyme/substrate (E/S) mix. The E/S mix contained buffer (KH2PO4), c-DNA-expressed P450 in insect cell microsomes, substrate (3-cyano-7-ethoxycoumarin (CEC) for CYP1A2 and CYP2C19, 7-methoxy-4-(trifluoromethyl)coumarin (7-MFC) for CYP2C9, 3-[2-(N,N-diethyl-N-methylammonium)ethyl]-7-methoxy-4-methylcoumarin (AMMC) for CYP2D6, 7-benzyloxytrifluoromethyl coumarin (7-BFC) and Dibenzylfluorescein (DBF) for CYP3A4) to give the final assay concentrations in a reaction volume of 200 µl. Reactions were terminated after different incubation times, depending on each cytochrome, by the addition of STOP solution (ACN/TrisHCl 0.5M 80:20 and NaOH 2N for CYP3A4 (DBF) and ACN/TrisHCl 0.5M 80:20 for the other cytochromes).

Fluorescence per well was measured using a fluorescence plate reader (Tecan M1000 pro), and the percentage of inhibition was calculated.

### 2.3. Animals

SAMP8 is an inbred mouse strain that has been generated by selective inbreeding of the AKR/J strain of mice [31,32]. It displays a phenotype of accelerated aging with behavioural abnormalities [33,34], the age-related cognitive decline [35], and several AD hallmarks [2,36]. Overall, it is widely used as a feasible rodent model of cognitive dysfunction and late-onset AD (LOAD) [37]. Senescence-Accelerated Mouse Resistant 1 (SAMR1) mouse is used as a healthy control mouse model.

**Figure 1:**
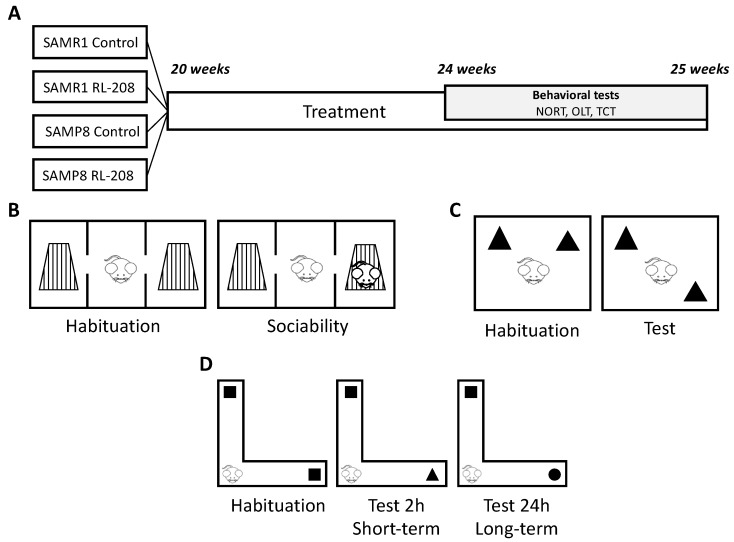
Scheme of experimental design (**A**), Scheme of Three Chamber Test (TCT) (**B**), Scheme of Object Location Test (OLT) (**C**), and scheme of Novel Object Recognition Test (NORT) (**D**).

Male SAMR1 and SAMP8 mice (n = 41) with 20-weeks-old were used to carry out cognitive and molecular analysis. The animals were randomly divided into four groups: SAMR1 control (SR1 Ct) group (n = 11), SAMP8 control (SP8 Ct) group (n = 8), SAMR1 treated with RL-208 (SR1 RL-208 (5 mg/Kg)) group (n = 10) and SAMP8 treated with RL-208 (SP8 RL-208 (5 mg/Kg)) group (n = 12). Animals had free access to food and water, under standard temperature conditions (22±2ºC) and 12h:12h light-dark cycles (300 lux/0 lux). RL-208, (3,4,8,9-tetramethyltetracyclo[4.4.0.0^3,9^.0^4,8^]dec-1-yl)-methylamine hydrochloride, (5 mg/kg/day) was administered through drinking water for four weeks before starting the cognitive test. RL-208 was administered up to euthanasia (Figure 1A). Water consumption was controlled each week for each cage and strain. Afterwards, RL-208 concentration was adjusted accordingly to reach the optimal dose for each cage.

All experimental procedures involving animals were performed followed by standard ethical guidelines European Communities Council Directive 86/609/EEC and by the Institutional Animal Care and Use Committee of the University of Barcelona (670/14/8102, approved at 11/14/2014) and by Generalitat de Catalunya (10291, approved 1/28/2018).

### 2.4. Behavioural and Cognitive Tests

#### 2.4.1. Three-Chamber Test 

The three-chamber test (TCT) was used to assess preference for social novelty (time spent with a novel intruder in contrast with a familiar one) and sociability (time spent with rodents) [38]. The apparatus consisted of a rectangular box with partitions separating the box into three chambers (Figure 1B). Each chamber was 15 × 15 × 20 cm with square openings. Testing occurs in a box with three equally dimensioned rooms. Each test consists of 15 minutes and is recorded with a camera. The animal is placed in the center of the box and allowed to explore the three chambers for 5 minutes. The time spent in each chamber was evaluated. Then, an intruder (same-sex and age) was added to one of the rooms in a metal cup, and behaviour is recorded for 10 minutes. The time spent in each chamber is evaluated as well as the time interacting with the intruder (e.g., sniffing, rears, entries in each chamber).

#### 2.4.2. Object Location Test 

The object location test (OLT) is a well-established task based on the spontaneous tendency of rodents to spend more time exploring a novel object location than a familiar object location, as well as to recognize when an object has been relocated [39]. The test was carried out for 3 days in a wooden box (50 × 50 × 25 cm), in which three walls were white except one that was black (Figure 1C). The first day, the box was empty, and the animals just habituated to the open field arena for 10 minutes. The second day, two objects were placed in front of the black wall, equidistant from each other and the wall. The objects were 10-cm high and identical. The animals were placed into the open field arena and allowed to explore both objects and surroundings for 10 minutes. Afterwards, animals were returned to their home cages, and the OLT apparatus was cleaned with 70% ethanol. On the third day, one object was moved in front of the white wall to test the spatial memory. Trials were recorded using a camera mounted above the open field area, and the total exploration time was determined by scoring the amount of time (seconds) spent sniffing the object in the new location (TN) and the object in the old location (TO). In order to evaluate the cognitive performance, the DI was calculated, which is defined as (TN-TO)/(TN+TO).

#### 2.4.3. Novel Object Recognition Test

The novel object recognition test (NORT) allows evaluating short- and long-term recognition memory involving cortical areas and the hippocampus [40,41]. The experimental apparatus used for this test was a 90º, two-arm, 25-cm-long, 20-cm-high, and a 5-cm-wide black maze of black polyvinyl chloride (Figure 1D). The walls could be removed for easy cleaning with 70% ethanol to eliminate olfactory cues, and light intensity in mid-field was 30 lux. The objects to be discriminated were made of plastic and chosen not to frighten mice and without any part likely to be bitten. Before performing the test, the mice were individually habituated to the apparatus for 10 min during 3 consecutive days. On day four the next day, the animals were allowed to explore freely a 10 min acquisition trial (First trial), during which they were placed in the maze in the presence of two identical novel objects (A+A’ or B+B’) at the end of each arm. The mouse was then removed from the apparatus and returned to its home cage. A 10 min retention trial (Second trial) was carried out two hours later. During this second trial, objects A and B were placed by to novel objects with different shapes and colors, and the mice were allowed to explore the maze for another 10 min. Twenty-four hours after the acquisition trial, the mice were tested again, with a new object and an object identical to the new one in the previous trial (B+C). The time that mice explored the novel object (TN) and time that mice explored the old object (TO) were measured from the video recordings from each trial session Exploration of an object was defined as pointing the nose towards the object at a distance ≤2 cms and/or touching it with the nose. Turning or sitting around the object was not considered exploration. To avoid object preference biases objects A and B were counterbalanced so that one-half of the animals in each experimental group were first exposed to object A and then to object B, whereas the other half first saw object B and then object A. To evaluate the cognitive performance, the discrimination index (DI) was calculated, which is defined as (TN-TO)/(TN+TO). 

### 2.5. Brain Processing

Three days after the behavioural and cognitive tests, mice were euthanized by cervical dislocation. Brains were immediately removed from the skull. The hippocampus and cortex were then isolated and frozen in powdered dry ice. They were maintained at −80 °C for further use. Tissue samples were homogenized in lysis buffer containing phosphatase and protease inhibitors (Cocktail II, Sigma). Total protein levels were obtained, and protein concentration was determined by the method of Bradford. 

### 2.6. Protein Level Determination by Western Blotting

For Western blotting (WB), aliquots of 15 μg of hippocampal protein extraction per sample were used. Protein samples were separated by Sodium dodecyl sulphate-Polyacrylamide gel electrophoresis (SDS-PAGE) (8–20%) and transferred onto Polyvinylidene difluoride (PVDF) membranes (Millipore). Afterwards, membranes were blocked in 5% non-fat milk in Tris-buffered saline (TBS) solution containing 0.1% Tween 20 TBS (TBS-T) for 1 hour at room temperature, followed by overnight incubation at a 4 °C with the primary antibodies listed in Table S1. Then, membranes were washed and incubated with secondary antibodies for 1 hour at room temperature. Immunoreactive proteins were viewed with the chemiluminescence-based detection kit, following the manufacturer’s protocol (ECL Kit, Millipore), and digital images were acquired using ChemiDoc XRS+ System (BioRad). Semi-quantitative analyses were performed using ImageLab software (BioRad), and results were expressed in arbitrary units (AU), considering control protein levels as 100%. Protein loading was routinely monitored by immunodetection of Glyceraldehyde-3-phosphate dehydrogenase (GAPDH). 

### 2.7. Detection of Oxidative Stress in the Hippocampus

H_2_O_2_ levels from cortex samples were measured as an indicator of oxidative stress, and it was quantified using the Fluorimetric Hydrogen Peroxide Assay Kit (Sigma) according to the manufacturer’s instructions. 

### 2.8. RNA Extraction and Gene Expression Determination

Total RNA isolation from hippocampal samples was carried out using TRIsure^TM^ reagent following the manufacturer’s instructions (Bioline, Meridian Bioscience Inc., UK). The yield, purity, and quality of RNA were determined spectrophotometrically with a NanoDrop^TM^ ND-1000 (Thermo Scientific, Wilmington, DE, USA) apparatus and an Agilent 2100B Bioanalyzer (Agilent Technologies, Palo Alto, CA, USA). RNAs with 260/280 ratios and RIN higher than 1.9 and 7.5, respectively, were selected. Reverse transcription-polymerase chain reaction (RT-PCR) was performed as follows: 2 μg of messenger RNA (mRNA) was reverse-transcribed using the High Capacity cDNA Reverse Transcription kit (Applied Biosystems, Foster City, CA, USA). Real-time quantitative PCR (qPCR) was used to quantify the mRNA expression of OS and synaptic plasticity genes listed in Table S2.

SYBR® Green real-time PCR was performed on a Step One Plus Detection System (Applied-Biosystems, Foster City, CA, USA) employing SYBR® Green PCR Master Mix (Applied-Biosystems, Foster City, CA, USA). Each reaction mixture contained 6.75 μL of complementary DNA (cDNA) (which concentration was 2 μg), 0.75 μL of each primer (which concentration was 100 nM), and 6.75 μL of SYBR® Green PCR Master Mix (2X). 

Data were analyzed utilizing the comparative cycle threshold (Ct) method (ΔΔCt), where the housekeeping gene level was used to normalize differences in sample loading and preparation [42]. Normalization of expression levels was performed with β-actin for SYBR® Green-based real-time PCR results. Each sample was analyzed in duplicate, and the results represent the n-fold difference of the transcript levels among different groups.

### 2.9. Measurement of proBDNF and mBDNF Protein Levels in the Hippocampus 

The hippocampal determination of pro-Brain-derived neurotrophic factor (proBDNF) and mature brain-derived neurotrophic factor (mBDNF) protein levels was performed using the enzyme-linked immunosorbent assay (ELISA) kit (Biosensis) according to the manufacturer’s instructions. 

### 2.10. Data Acquisition and Statistical Analysis 

Behavioural analysis was performed blindly, the person who evaluated videos was different from the person who made the behavioural tests. Furthermore, videos are named with a blind code to avoid analysis bias. Data analysis was conducted using GraphPad Prism ver. 7 statistical software. Data are expressed as the mean ± standard error of the mean (SEM) of at least 5 samples per group. Strain and treatment effects were compared using the two-way analysis of variance (ANOVA), followed by Tukey post-hoc analysis or two-tail student’s t-test when it was necessary. Statistical significance was considered when p-values were <0.05. The statistical outliers were determined with Grubbs’ test and when necessary were removed from the analysis. 

## 3. Results

### 3.1. In Vitro Microsomal Stability and Cytochrome Inhibition

RL-208 was further studied in vitro for ascertaining their microsomal stability and CYP inhibition. RL-208 showed good microsomal stability in rat and mice microsomes, and did not inhibit in a significant way cytochromes CYP2C9, 12 ± 2%, CYP2D6 10 ± 3%; CYP1A2, 5 ± 2%; CYP2C19, 23 ± 1%; CYP3A4 (BFC), 35 ± 4%; CYP3A4(DBF), 3 ± 1%). 

### 3.2. Improvement on Social Behaviour and Cognition after Treatment with RL-208 

**Figure 2:**
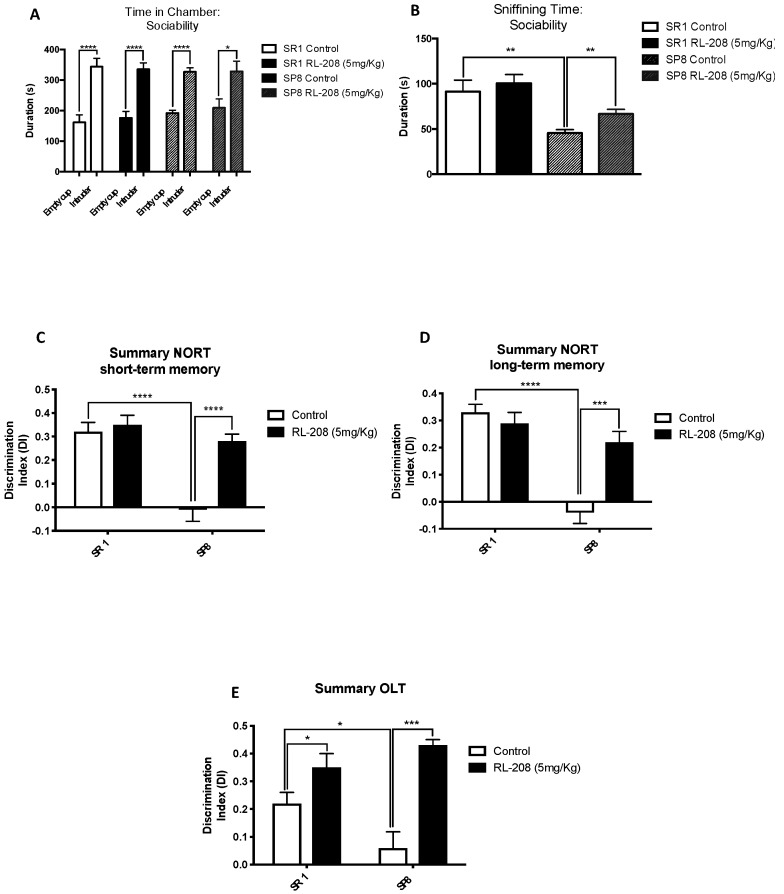
Results of Three Chamber Test (TCT), Object Location Test (OLT), and Novel Object Recognition Test (NORT) in male mice at 24-weeks-old SR1 and SP8 Ct mice groups and SR1 and SP8 treated with RL-208 (5 mg/Kg) mice groups. For TCT: Time spent in the chamber (**A**) and sniffing time: sociability with the intruder animal (**B**). For NORT: Summary of Discrimination Index (DI) from short-term memory (**C**), and summary of DI from long-term memory (**D**). For OLT: Summary of DI (**E**). Values represented are mean ± Standard error of the mean (SEM); n = 41 (SR1 Ct n = 11; SP8 Ct n = 8; SR1 RL-208 n = 10; SP8 RL-208 n = 12). * *p* < 0.05; ** *p* < 0.01; *** *p* < 0.001; **** *p* < 0.0001.

The TCT assess the general sociability in mice. In the sociability phase, in all experimental groups, the presence of an intruder increased significantly the time spent in the intruder chamber instead of the empty cup chamber (Figure 2A). Moreover, the time sniffing the intruder mouse was significantly higher in the SR1 Ct group compared to the SP8 Ct group, confirming the behavioural abnormalities described in the SAMP8 mouse model. After RL-208 treatment, the time sniffing the intruder was significantly augmented in SAMP8 strain, proving the beneficial effects on social behaviour (Figure 2B; Table S3). 

NORT evaluation confirmed the cognitive impairment of the SAMP8 mouse model in both short- and long-term recognition memories in comparison with the SAMR1 (Figure 2C,D; Table S4). Strikingly, the SP8 RL-208 group exhibited a significant gain in both short- and long-term recognition memories compared to the SP8 Ct group, obtaining significant higher DI values (Figure 2C,D). Conversely, no significant changes in both short- and long-term DI values between SR1 groups were found. Regarding OLT evaluation, a significantly higher DI value in the SR1 Ct group compared to the SP8 Ct mice group was found (Figure 2E). Likewise, a better spatial memory in both treated mice groups was found, showing significant higher DI values after RL-208 treatment (Figure 2E; Table S5).

### 3.3. Changes in NMDAR and Apoptotic Pathways Induced by RL-208 

**Figure 3:**
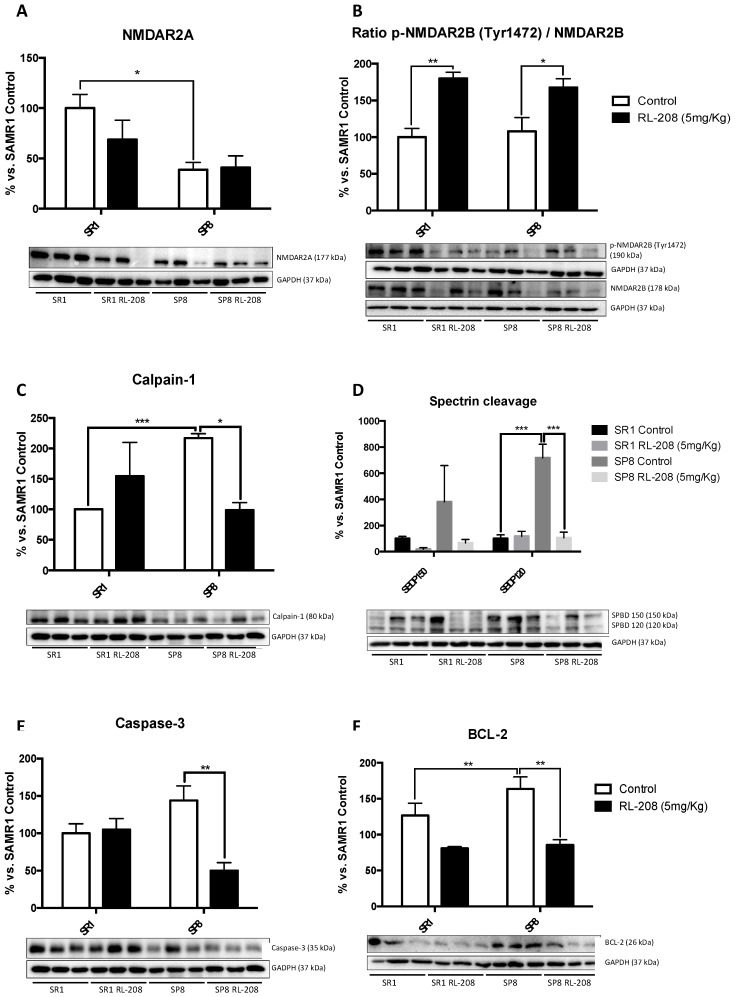
Representative Western Blot and quantifications for NMDAR2A (**A**), the ratio of p-NMDA2B/NMDAR2B (**B**), Calpain-1 (**C**), ratio SBPD/Spectrin (**D**), Caspase-3 (**E**), BCL-2 (**F**). Values in bar graphs are adjusted to 100% for protein levels of the control SAMR1 (SR1 Ct). Values are the mean ± Standard error of the mean (SEM); (n = 6 for each group). * *p* < 0.05; ** *p* < 0.01; *** *p* < 0.001.

NMDAR changes and apoptotic markers were studied as a learning memory and synaptic plasticity activation. A significant decrease in NMDAR2A protein level was found in SP8 Ct compared to the SR1 Ct (Figure 3A), suggesting its participation in the cognitive decline presented by the SAMP8 mouse model. However, RL-208 treatment did not produce significant differences in the NMDAR2A protein levels neither in SR1 nor in SP8. Interestingly, RL-208 increased in a significant way p-NMDAR (Tyr1472) protein levels in both strains (Figure 3B), suggesting an improvement in neuronal functionality.

Next, we evaluated the effects of RL-208 on the proteolytic processes that lead to apoptosis. Calpain-1 and 150 Spectrin Breakdown Products (SBDP) protein levels increased in SP8 Ct in comparison with the SR1 Ct group. RL-208 treatment reduced Calpain-1, Caspase-3 and 120BPD in the SP8 strain, not in SR1 (Figure 3C–E).

By contrast, B-cell lymphoma-2 (BCL-2) protein levels diminished, only reaching significance in the SP8 mouse model (Figure 3F). 

### 3.4. Increased Neurotrophins and Synaptic Markers Protein Levels after Treatment with RL-208

**Figure 4:**
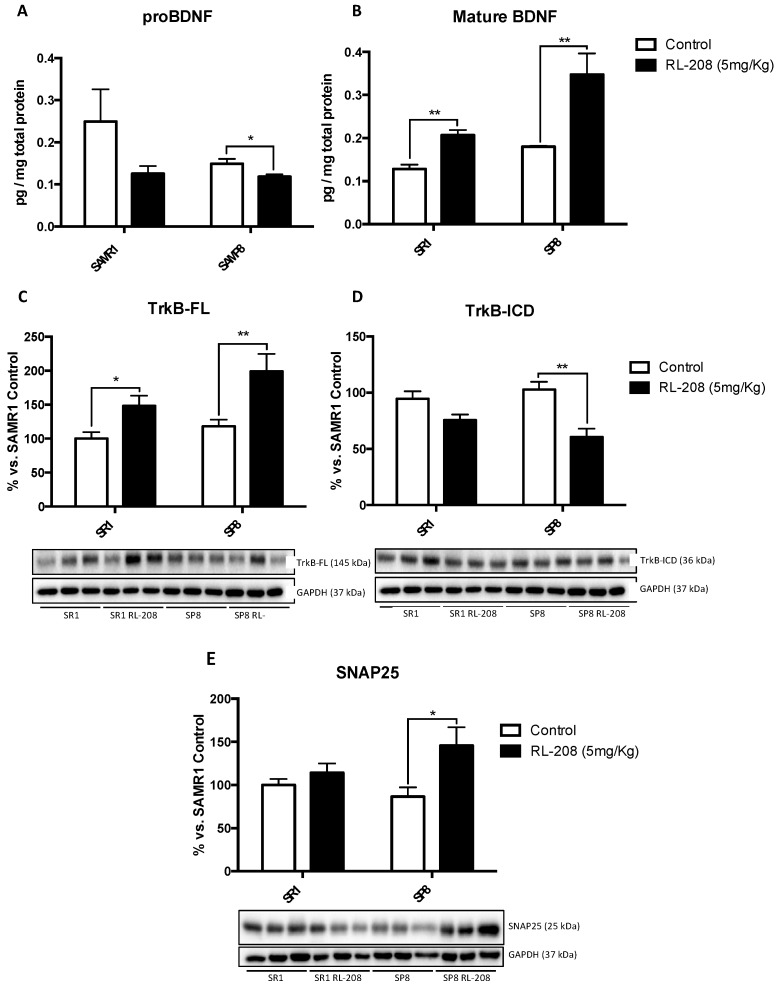
Protein levels of proBDNF (**A**), and mBDNF (**B**). Representative Western Blot and quantifications for TrkB-FL (**C**), TrkB-ICD (**D**) and SNAP25 (**E**). Values represented are mean ± Standard error of the mean (SEM); (n = 6 for each group). * *p* < 0.05; ** *p* < 0.01; *** *p* < 0.001.

A significant reduction in proBDNF protein levels in SP8 treated mice compared to the control group were found. Differences in SR1 strain were not significant (Figure 4A). Conversely, a significant augment in mature BDNF protein levels in RL-208 treated mice compared to control groups were found (Figure 4B). Tropomyosin-related kinase B full-length (TrkB-FL) protein levels increased significantly both in SR1 and SP8 treated with RL-208 (Figure 4C), whereas reduced TrkB intracellular fragment (TrkB-ICD) protein levels were observed (Figure 4D). TrkB signaling pathway regulates synaptosomal-associated protein 25 (SNAP25), a synaptic plasticity marker, and as expected, significant-high SNAP25 protein levels were found in SP8 RL-208 (Figure 4E). 

**Figure 5:**
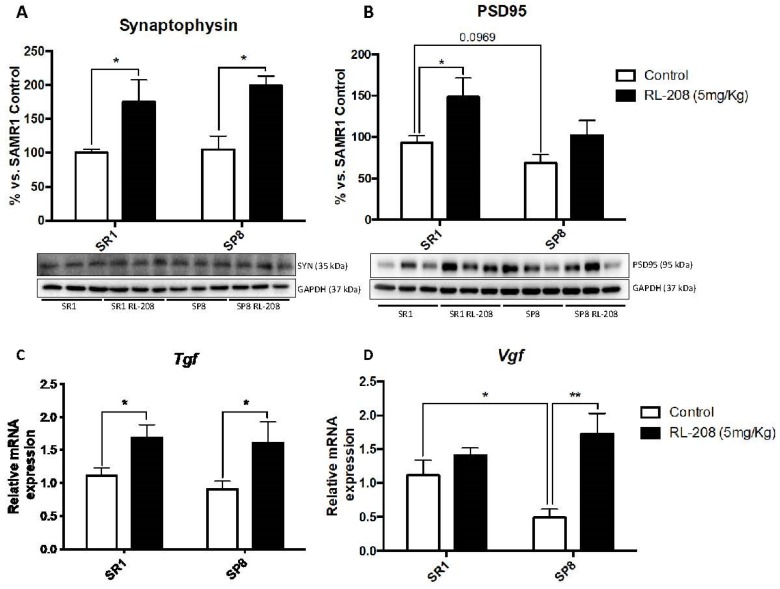
Representative Western Blot and quantifications for Synaptophysin (**A**), and PSD95 (**B**). Representative gene expression for *Tgf* (**C**), and *Vgf* (**D**). Values in bar graphs are adjusted to 100% for protein levels of the control SAMR1 (SR1 Ct). Gene expression levels were determined by real-time PCR. Values are the mean ± Standard error of the mean (SEM); (n = 6 for each group). * *p* < 0.05; ** *p* < 0.01.

Deeping on synaptic plasticity markers, a significant increase in Synaptophysin (SYN) protein levels were found in RL-208 treated mice (Figure 5A). Postsynaptic density protein 95 (PSD95) protein levels were also augmented but did not reach significance in SP8 mice (Figure 5B). Regarding the neurotrophic factors, a significant gain in gene expression of the tumor growth factor (*Tgf*) in RL-208 treated mice groups in comparison with the control groups, was observed (Figure 5C). Likewise, a significant increase in gene expression of VGF nerve growth factor inducible (*Vgf*) in SP8 RL-208 compared to the SP8 Ct group, but no changes between SR1 mice groups were found (Figure 5D).

### 3.5. Changes in Protein Levels and Gene Expression of Antioxidant and Pro-oxidant Enzymes and ROS Levels after Treatment with RL-208

**Figure 6:**
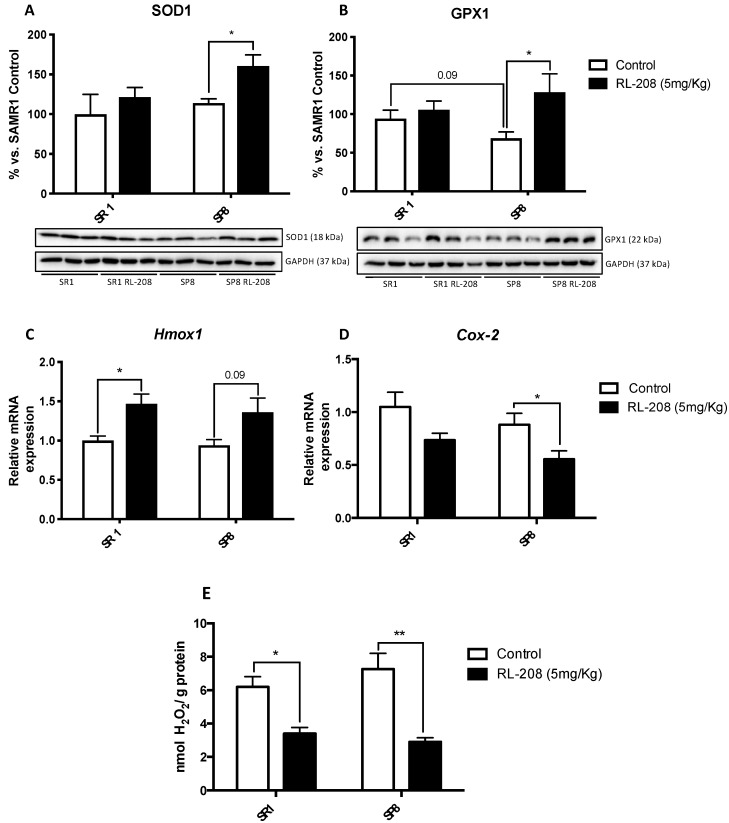
Representative Western Blot and quantifications of antioxidant enzymes for SOD1 (**A**), and GPX1 (**B**). Representative gene expression of the antioxidant enzyme for *Hmox1* (**C**), and pro-oxidant enzyme for *Cox2* (**D**). Representative OS measured as hydrogen peroxide concentration in homogenates of the hippocampus tissue (**E**). Values in bar graphs are adjusted to 100% for protein levels of the control SAMR1 (SR1 Ct). Gene expression levels were determined by real-time PCR. Values represented are mean ± Standard error of the mean (SEM); (n = 6 for each group). * *p* < 0.05; ** *p* < 0.01.

RL-208 increased, in a significant way, Superoxide Dismutase 1 (SOD1) and Glutathione Peroxidase 1 (GPX1), antioxidant protein levels SP8 mice, but not in SR1 mice (Figure 6A,B). Moreover, GPX1 protein levels tended to decrease in the SP8 Ct group compared to the SAMR1 Ct group was observed (Figure 6B). RL-208 elevated gene expression of Heme oxygenase decycling 1 (*Hmox1*), an important key enzyme in cellular antioxidant-defense in treated mice (Figure 6C). Conversely, RL-208 decreased gene expression of Cyclooxygenase-2 (*Cox2*) in treated mice groups in comparison with the control groups, being significant in the SP8 strain (Figure 6D). Finally, the evaluation of the hydrogen peroxide levels in the hippocampus showed a significant decrease in reactive oxygen species (ROS) levels in both RL-208 treated mice groups compared to the control groups (Figure 6E). 

### 3.6. Changes in CDK5/p25-35 Pathway Activation and Tau Phosphorylation after Treatment with RL-208

**Figure 7:**
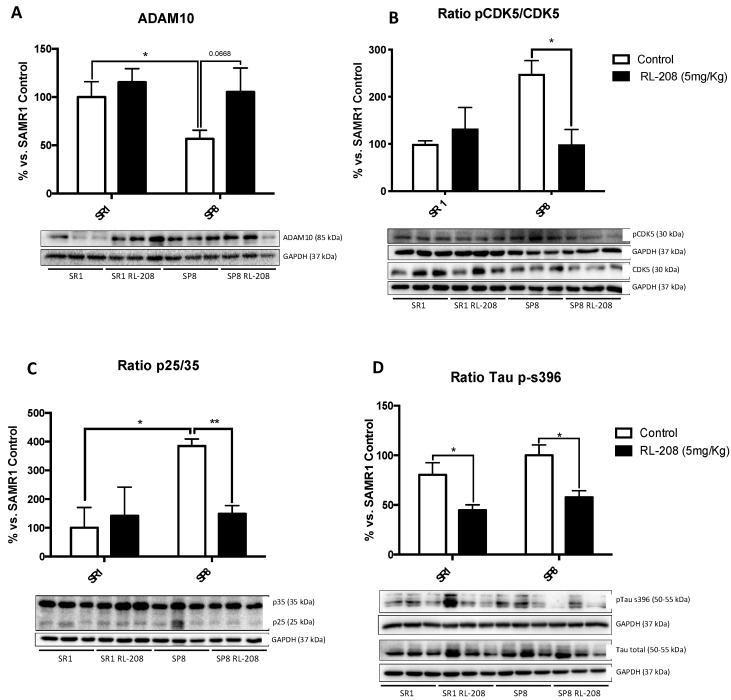
Representative Western Blot and quantifications for ADAM10 (**A**), ratio p-CDK5/CDK5 (**B**), ratio p25/p35 (**C**), and ratio Tau (p-s396) (**D**). Values in bar graphs are adjusted to 100% for protein levels of the control SAMR1 (SR1 Ct). Values represented are mean ± Standard error of the mean (SEM); (n = 6 for each group). * *p* < 0.05; ** *p* < 0.01.

ADAM10 protein levels were diminished in SAMP8 in reference to SAMR1, and RL-208 prevented the loss of this secretase (Figure 7A). The Cyclin-Dependent Kinases 5 (CDK5)/p25-p35 and Tau phosphorylation were evaluated by WB (Figure 7B,C). We found a significant high p25/p35 ratio in the SP8 Ct compared to the SR1 Ct, accordingly with the increase in calpain activity described above. Likewise, CDK5 activation (measured by p-CDK5/CDK5 ratio) and p25/p35 ratio were significantly reduced in the SP8 RL-208 group compared to the control group (Figure 7B,C). Considering these results, we evaluated Tau hyperphosphorylation protein levels. A significant reduction in Tau phosphorylation in both treated mice groups was found, specifically for the Ser396 phosphorylation site (Figure 7D).

## 4. Discussion

As aforementioned, the alteration of NMDAR has been associated with neurodegenerative disorders, such as AD [43]. It has been well documented that competitive pharmacological blockade of NMDAR functions leads to cognitive disability [27] and impaired neuroplasticity [44], increasing apoptotic neuronal death [45]. However, memantine has demonstrated specific effects because its low-affinity, uncompetitive antagonist performance, and can block the NMDAR over-activation without affecting its normal activation [46,47]. The current study provides pieces of evidence demonstrating that the novel non-competitive NMDAR antagonist RL-208 can be a promising therapy for age-related cognitive and AD.

Early preclinical in vitro experiments indicated that RL-208 displays low IC_50_, by 1 μM [30], has low metabolism, and did not interact with cytochromes, having then a good druggable profile to test it in in vivo models and to reach the proof of concept of in vivo effectivity. 

Several preclinical studies in different transgenic mice models of AD with NMDAR antagonists, including memantine, demonstrate beneficial cognitive activities [28,48,49,50,51,52]. In line with these findings, RL-208 treatment improved social behaviour and restoring cognitive impairment in SAMP8 animals, by using TCT and memory test (NORT and NOLT) respectively. As expected, we did not find any substantial improvements in behaviour and cognition in the control strain SAMR1 after RL-208 treatment, indicating that uncompetitive antagonist is effective in front overstimulation of NMDAR but not in physiological conditions. 

The behavioural and cognitive changes induced by RL-208 were accompanied by changes in several molecular pathways associated with NMDAR functionality. RL-208 increased p-NMDAR2B (Tyr1472), which has been proved to play crucial roles in the induction of long-term potentiation (LTP), and the hippocampus-dependent memory formation [53]. There are several molecular pathways related to apoptosis that participated in cellular physiological and pathological processes, i.e., proteases as Calpain-1 and Caspase, or specific mediators as BCL-2. RL-208 decreased activation and protein levels of Calpain-1 and Caspase-3 proteases and decreased BCL-2 protein levels. Interestingly, Caspase-3 and BCL-2 changes were significant in SAMP8 treated with RL-208, confirming that RL-208 works under the overactivation of the NMDAR. Lack of effect in healthy conditions turn out in fewer side effects. To the best of our knowledge, there is only one report using memantine at a similar dose (4 mg/kg) which shows similar antiapoptotic effects related to NMDAR antagonism in the rats’ hippocampus [54].

Previous studies have been described as the ability of NMDAR antagonists to modify synaptic plasticity [55,56,57]. In our hands, RL-208 treatment increased the majority of the synaptic plasticity markers such as SYN, PSD-95, and *Tgf* in the SAMP8 and SAMR1 hippocampus pointed out that the improvement in cognition under pathological conditions was associated with hippocampal plasticity. In the same line, neurotrophic signaling is severely impaired in AD, being BDNF/TrkB representative signaling pathway altered. BDNF/TrKB signaling pathway was ameliorated under RL-208 treatment, with significant increases in mBDNF, TrKB-FL, and SNAP25 protein levels. These results could indicate that RL-208 contributes to restoring cognition in SAMP8 mouse because of the increase in BDNF/TrKB signaling. 

It has been reported that OS is involved in several neurodegenerative disorders, mediating neuronal death [58]. Several reports suggest that NMDAR activation mediated OS, causing synapse alterations [59,60,61,62]. Concretely, ROS leads to neuronal alterations and ultimately produce synaptic dysfunction, which is a critical factor of the age-related cognitive decline and AD [63]. RL-208 decrease hippocampal ROS levels in treated mice. In parallel, SOD1 and GPX1 protein levels and *Hmox1* gene expression increased, as well as *Cox-2* gene expression diminished in SAMP8 after RL-208 treatment. Then, in line with our findings, RL-208 delivered a significant diminution in OS because of its role as uncompetitive NMDAR antagonist.

Tau hyperphosphorylation is a characteristic histological mark in several neurodegenerative disorders. Tau phosphorylation levels are regulated by a complex network of protein kinases and phosphatases, such as CDK5 [64]. RL-208 reduced the activity of the CDK5 in SAMP8 mice, where CDK5 is overactivated. Furthermore, a significant reduction in the p25/p35 ratio in SP8 RL-208 group. Interestingly CDK5 co-activator p25 is cleaved by Calpain-1 from p35 peptide harbored in the cytoplasmic membrane, and as mentioned RL-208 reduced Calpain-1 proteolytic activity. Accordingly, significant diminution of p-Tau [Ser396] was found after RL-208 administration. To our knowledge, memantine is also described as able to reduce tau phosphorylation [65,66,67], linking the RL-208 antagonist action on NMDAR again with the neuroprotectant effectivity in SAMP8. 

**Figure 8:**
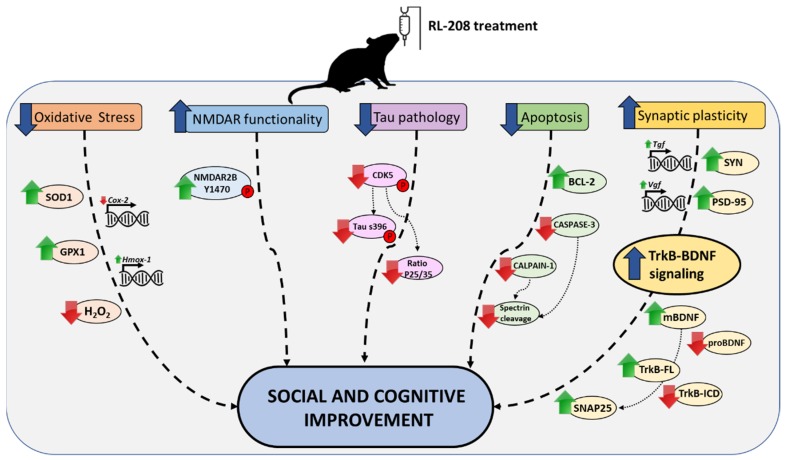
Illustrative cartoon of molecular and cognitive effects after RL-208 treatment.

In sum, our in vivo study in SAMP8 demonstrated the therapeutic potential of RL-208, a novel NMDAR uncompetitive antagonist, for age-related cognitive decline, and AD. Because it is of interest to shed light on the underlying mechanisms by which NMDAR mediates neuroprotection and new pharmacological approaches are needed to fight devastating neurodegenerative diseases as AD, RL-208 beneficial effects described here, offer new clues to face the challenge in drug discovery (Figure 8). Noteworthy, more studies are needed to draw the complete landscape action for NMDAR antagonist, and in particular for RL-208 to proceed towards translational clinical application.

## Supplementary Materials

The following are available online at https://www.mdpi.com/1999-4923/12/3/284/s1, Table S1: Antibodies used in Western blot studies, Table S2: Primers used in qPCR studies. Table S3: Parameters measured in the Three Chamber. Test Table S4: Parameters measured in the Novel object recognition test (NORT); Table S5: Parameters measured in the Object location test (OLT).
