# Supplementary material for: A Novel NMDA Receptor Antagonist Protects against Cognitive Decline Presented by Senescent Mice
Source: Pharmaceutics. 2020 Mar 22;12(3):284. doi: 10.3390/pharmaceutics12030284 (PMC7151078; doi:10.3390/pharmaceutics12030284)
Supplement: Supplementary file 1 [file pharmaceutics-12-00284-s001.zip › pharmaceutics-738871-suppl/Table S1.docx]

**Table S1.** Antibodies used in Western blot studies.

| Antibody | Host | Source/Catalog | WB dilution |
| --- | --- | --- | --- |
| **NMDAR2A** | Mouse | Santa Cruz/sc-515148 | 1:1000 |
| **NMDAR2B** | Mouse | Santa Cruz/sc-365597 | 1:1000 |
| **p-NMDAR2B (Tyr1472)** | Rabbit | Invitrogen/OPA1-04116 | 1:1000 |
| **Calpain-1** | Mouse | BioRad/AHP2443 | 1:1000 |
| **Spectrin** | Mouse | Millipore/MAB1622 | 1:1000 |
| **Caspase-3** | Rabbit | BD Transduction Laboratories/C31720 | 1:1000 |
| **BCL-2** | Rabbit | Cell Signaling/#2870 | 1:1000 |
| **SYN** | Rabbit | Dako/CloneSY38 | 1:2000 |
| **PSD95** | Rabbit | Abcam/ab18258 | 1:1000 |
| **TrkB** | Rabbit | Santa Cruz/sc-8316 | 1:1000 |
| **SNAP25** | Mouse | Santa Cruz/sc-20038 | 1:1000 |
| **SOD1** | Mouse | Calbiochem/574597 | 1:1000 |
| **GPX1** | Rabbit | Novus Biological/NBP1-33620 | 1:1000 |
| **p35/p25** | Rabbit | Cell Signaling/#C64B10 | 1:1000 |
| **p-Tau Ser396** | Rabbit | Invitrogen/44752G | 1:1000 |
| **Tau total** | Goat | Santa Cruz/sc-1995 | 1:1000 |
| **CDK5** | Rabbit | Santa Cruz/sc-173 | 1:1000 |
| **p-CDK5** | Rabbit | Abcam/ab63550 | 1:1000 |
| **GAPDH** | Mouse | Millipore/MAB374 | 1:2000 |
| **Goat-anti-mouse HRP conjugated** |  | BioRad/170-5047 | 1:2000 |
| **Goat-anti-rabbit HRP conjugated** |  | BioRad/170-6515 | 1:2000 |
| **Donkey-anti-goat HRP conjugated** |  | Santa Cruz/sc-2020 | 1:2000 |
