# Supplementary material for: A Novel NMDA Receptor Antagonist Protects against Cognitive Decline Presented by Senescent Mice
Source: Pharmaceutics. 2020 Mar 22;12(3):284. doi: 10.3390/pharmaceutics12030284 (PMC7151078; doi:10.3390/pharmaceutics12030284)
Supplement: Supplementary file 1 [file pharmaceutics-12-00284-s001.zip › pharmaceutics-738871-suppl/Table S2.docx]

| Target | Product size (bp) | Forward primer (5’-3’) | Reverse primer (5’-3’) |
| --- | --- | --- | --- |
| *Hmox1* | 177 | TGACACCTGAGGTCAAGCAC | GTCTCTGCAGGGGCAGTATC |
| *Cox-2* | 126 | TGACCCCCAAGGCTCAAATA | CCCAGGTCCTCGCTTATGATC |
| *Vgf* | 178 | GTCAGACCCATAGCCTCCC | CTCGGACTGAAATCTCGAAGTTC |
| *Tgf* | 204 | CAGGGTGAAGGGGAAAACTC | AGTTCGGTCATTCAGTCTCGC |
| *β-Actin* | 190 | CAACGAGCGGTTCCGAT | GCCACAGGTTCCATACCCA |

**Table S2.** Primers used in qPCR studies.
