# Supplementary material for: A Novel NMDA Receptor Antagonist Protects against Cognitive Decline Presented by Senescent Mice
Source: Pharmaceutics. 2020 Mar 22;12(3):284. doi: 10.3390/pharmaceutics12030284 (PMC7151078; doi:10.3390/pharmaceutics12030284)
Supplement: Supplementary file 1 [file pharmaceutics-12-00284-s001.zip › pharmaceutics-738871-suppl/Table S3.docx]

**Table 3.** Parameters measured in the Three Chamber Test (TCT). (sec): Time in each chamber. Results are expressed as a mean ± Standard error of the mean (SEM). $ p <0.05 vs SR1 RL-208.

| Time in Chamber: Habituation | SR1 Control | SR1 RL-208 (5mg/Kg) | SP8 Control | SP8 RL-208 (5mg/Kg) |
| --- | --- | --- | --- | --- |
| Right zone | 109.30 ± 20.05 | 86.72 ± 17.61 | 119.4 ± 8.48 | 149.10 ± 10.41^$^ |
| Left zone | 110.09 ± 22.69 | 108.57 ± 23.52 | 119.21 ± 9.33 | 97.55 ± 9.68 |
| Center zone | 80.62 ± 11.20 | 104.71 ± 17.62 | 51.39 ± 2.13^$^ | 53.35 ± 6.20 |
