# Supplementary material for: A Novel NMDA Receptor Antagonist Protects against Cognitive Decline Presented by Senescent Mice
Source: Pharmaceutics. 2020 Mar 22;12(3):284. doi: 10.3390/pharmaceutics12030284 (PMC7151078; doi:10.3390/pharmaceutics12030284)
Supplement: Supplementary file 1 [file pharmaceutics-12-00284-s001.zip › pharmaceutics-738871-suppl/Table S4.docx]

**Table 4.** Parameters measured in the Novel object recognition test (NORT). (sec): Time spent exploring each object during the familiarization phase. Results are expressed as a mean ± Standard error of the mean (SEM). *p <0.05 vs SR1 Control.

| Time spent exploring objects (sec): Familiarization phase | SR1 Control | SR1 RL-208 (5mg/Kg) | SP8 Control | SP8 RL-208 (5mg/Kg) |
| --- | --- | --- | --- | --- |
| Left object | 42.11 ± 4.25 | 39.88 ± 4.06 | 40.15 ± 3.55 | 39.63 ± 4.29 |
| Right Object | 40.56 ± 3.21 | 41.08 ± 2.56 | 42.36 ± 5.33 | 41.27 ± 3.07 |
