# Supplementary material for: A Novel NMDA Receptor Antagonist Protects against Cognitive Decline Presented by Senescent Mice
Source: Pharmaceutics. 2020 Mar 22;12(3):284. doi: 10.3390/pharmaceutics12030284 (PMC7151078; doi:10.3390/pharmaceutics12030284)
Supplement: Supplementary file 1 [file pharmaceutics-12-00284-s001.zip › pharmaceutics-738871-suppl/Table S5.docx]

**Table 5.** Parameters measured in the Object location test (OLT). (sec): Time spent exploring each object. Results are expressed as a mean ± Standard error of the mean (SEM). *p <0.05 vs SR1 Control.

| Time spent exploring objects (sec): Habituation phase | SR1 Control | SR1 RL-208 (5mg/Kg) | SP8 Control | SP8 RL-208 (5mg/Kg) |
| --- | --- | --- | --- | --- |
| Object A1 | 28.01 ± 2.45 | 21.96 ± 1.96 | 33.35 ± 3.86 | 23.13 ± 2.09 |
| Object A2 | 29.43 ± 2.43 | 25.78 ± 3.06 | 29.96 ± 4.23 | 26.47 ± 2.87 |
